# A clinical, proteomics, and artificial intelligence‐driven model to predict acute kidney injury in patients undergoing coronary angiography

**DOI:** 10.1002/clc.23143

**Published:** 2019-01-08

**Authors:** Nasrien E. Ibrahim, Cian P. McCarthy, Shreya Shrestha, Hanna K. Gaggin, Renata Mukai, Craig A. Magaret, Rhonda F. Rhyne, James L. Januzzi

**Affiliations:** ^1^ Cardiology Division Massachusetts General Hospital Boston Massachusetts; ^2^ Harvard Medical School Boston Massachusetts; ^3^ Prevencio, Inc. Kirkland Washington; ^4^ Baim Institute for Clinical Research Boston Boston Massachusetts

**Keywords:** coronary angiography, kidney injury, risk prediction, risk score

## Abstract

**Background:**

Standard measures of kidney function are only modestly useful for accurate prediction of risk for acute kidney injury (AKI).

**Hypothesis:**

Clinical and biomarker data can predict AKI more accurately.

**Methods:**

Using Luminex xMAP technology, we measured 109 biomarkers in blood from 889 patients prior to undergoing coronary angiography. Procedural AKI was defined as an absolute increase in serum creatinine of ≥0.3 mg/dL, a percentage increase in serum creatinine of ≥50%, or a reduction in urine output (documented oliguria of <0.5 mL/kg per hour for >6 hours) within 7 days after contrast exposure. Clinical and biomarker predictors of AKI were identified using machine learning and a final prognostic model was developed with least absolute shrinkage and selection operator (LASSO).

**Results:**

Forty‐three (4.8%) patients developed procedural AKI. Six predictors were present in the final model: four (history of diabetes, blood urea nitrogen to creatinine ratio, C‐reactive protein, and osteopontin) had a positive association with AKI risk, while two (CD5 antigen‐like and Factor VII) had a negative association with AKI risk. The final model had a cross‐validated area under the receiver operating characteristic curve (AUC) of 0.79 for predicting procedural AKI, and an in‐sample AUC of 0.82 (*P* < 0.001). The optimal score cutoff had 77% sensitivity, 75% specificity, and a negative predictive value of 98% for procedural AKI. An elevated score was predictive of procedural AKI in all subjects (odds ratio = 9.87; *P* < 0.001).

**Conclusions:**

We describe a clinical and proteomics‐supported biomarker model with high accuracy for predicting procedural AKI in patients undergoing coronary angiography.

## 1 INTRODUCTION

The incidence of acute kidney injury (AKI) following angiographic procedures varies widely because of different definition criteria. Furthermore, the presence of comorbidities including diabetes, chronic kidney disease (CKD), and heart failure (HF) further increase the risk of AKI development.^1^ Causes of peri‐procedural AKI after angiographic procedures include contrast‐induced AKI and, less commonly, atheroembolism. Regardless of cause, AKI has substantial impact on patient management and prognosis; it has been associated with worsening of CKD, requirement for dialysis, prolonged hospital stay, and higher mortality rates and healthcare costs.^2^ Development of AKI is diagnosed using changes in serum creatinine or estimated glomerular filtration rate (eGFR). However, these measures of kidney function are only modestly useful for accurate prediction of risk for kidney injury.^3^ This has led to interest in developing tools to accurately prospectively predict incident AKI and in some cases, earlier than when changes in creatinine or eGFR may occur.^4^, ^5^, ^6^ In recent studies, machine learning was employed to develop models that predicted AKI in hospitalized patients with excellent accuracy;^7^, ^8^ and similarly, genomic and proteomic characterization of AKI has been undertaken with varying results.^9^, ^10^, ^11^ To the best of our knowledge, machine learning for prediction of AKI in patients undergoing coronary angiography has not yet been studied. As such, we hypothesized that a proteomics‐based and artificial intelligence‐driven biomarker approach together with clinical risk factors would predict procedural AKI risk in patients enrolled in the Catheter Sampled Blood Archive in Cardiovascular Diseases (CASABLANCA) undergoing coronary angiographic procedures with or without interventions for various acute and non‐acute indications.

## 2 METHODS

All study procedures were approved by the Partners Healthcare Institutional Review Board and carried out in accordance with the Declaration of Helsinki.

The design of the CASABLANCA (NCT NCT00842868) study has been detailed previously.^12^ Briefly, 1251 patients undergoing coronary and/or peripheral angiography with or without intervention between 2008 and 2011 were prospectively enrolled at the Massachusetts General Hospital in Boston, Massachusetts. Patients were referred for angiography for various acute and non‐acute indications. Of the 1251 patients enrolled, we excluded patients who did not undergo a coronary angiogram, patients who had a history of renal replacement therapy, those with missing blood urea nitrogen or creatinine values, and those with an insufficient quantity of sample. This left us with 889 patients undergoing coronary angiography with available blood samples.

After informed consent was obtained, detailed clinical and historical variables were recorded using a standardized case report form at the time of the angiographic procedure. This case report form included more than 100 clinical variables acquired at the time of study entry as well as results of coronary angiography. Angiographic results were based on visual interpretation by the operator, verified through the catheterization report.

Median follow‐up was 4 years, with a maximum follow‐up of 6 years. Follow‐up was complete for all patients. Processes for identification and adjudication of clinical endpoints were as previously described^12^ and included review of medical records, as well as phone follow‐up with patients and/or managing physicians and was performed by physicians blinded to biomarker concentrations. The Social Security Death Index and/or postings of death announcements were used to confirm vital status. A detailed definition of endpoints for CASABLANCA was previously published.^12^

Specific to this analysis, procedural AKI was defined as an abrupt reduction in kidney function with an absolute increase in serum creatinine of more than or equal to 0.3 mg/dL, a percentage increase in serum creatinine of ≥50%, or a reduction in urine output (documented oliguria of <0.5 mL/kg per hour for >6 hours), within 7 days after contrast exposure.

Baseline characteristics between those who developed procedural AKI and those who did not were compared. Dichotomous variables were compared using Fishers exact test, while continuous variables were compared using *t* test or Wilcox Rank sum test.

A total of 15 mL of blood was obtained immediately before the angiographic procedure through a centrally‐placed vascular access sheath. The blood was immediately centrifuged for 15 minutes, serum and plasma aliquoted on ice, and frozen at −80°C until biomarker measurement. The samples for the present study were analyzed after the first freeze‐thaw cycle for baseline biomarker values only. Luminex xMAP technology, is a bead‐based multiplexed immunoassay system in a microplate format. The multiplexed assays were developed by Myriad RBM at their Austin, Texas facility. Each analyte assay was individually designed in a single assay format. The individual assays were validated according to CLSI Standards and thoroughly tested at the simplex stage before multiplexing. After multiplexing key performance parameters, such as LLOQ, LDD, and precision were established prior to every kit release. During the assay runs, laboratory information management system (LIMS) provided chain of custody and data logging information for samples throughout the testing process. Sample plating was verified by two technicians and run in temperature‐controlled lab. Native controls were run in duplicate alongside samples. Standard curves were at the front and back of each plate to minimize between and within run impression. All samples and reagent handling were automated. A minimum of 50 beads were analyzed per analyte and a 8‐point standard curve fitting with advanced algorithms ensured accuracy for sample concentrations. Controls followed Westgard rules to monitor unwanted trending. Sample results were manually reviewed before release. The data was backed up on site with long‐term off‐site storage. We measured 109 biomarkers in blood (Supporting Information, Table S1) from 889 patients undergoing coronary angiographic procedures for various indications.

A complete case analysis was performed; blood urea nitrogen, or creatinine values were missing with some patients (n = 167), so these patients were removed from the analysis. One other patient was removed from the analysis for having an insufficient quantity of sample, leaving 889 samples available for analysis. For any biomarker result that was below the limit of detection, we utilized a standard approach of imputing concentrations 50% below the limit of detection.

To facilitate the machine learning analysis, the concentrations for all proteins underwent the following transformations: (a) they were log‐transformed to achieve a normal distribution, (b) outliers were clipped at the value of three times the median absolute deviation, and (c) the values were re‐scaled to a distribution with zero mean and unit variance. The starting sets of variables consisted of all 109 proteins, as well as clinical factors in the CASABLANCA dataset that were chosen for their possible clinical relevance. Clinical and biomarker predictors of AKI were identified using least‐angle regression. In this method, factors were included in the model one at a time, with their coefficients determined by their correlation with the outcome. This was repeated until all factors were included in the model, and the step at which the performance plateaued resulted in our initial panel of interest. Starting with this panel of interest, predictive analyses were run on the training set using least absolute shrinkage and selection operator (LASSO) with logistic regression, predicting the outcome of procedural AKI using only the variables in the panel of interest. This model‐development process was done through Monte Carlo cross‐validation, using 400 iterations with an 80:20 (training: test) split. If the performance of the least contributing variable in the panel was not statistically significant, it was removed from the panel and the analysis repeated until the predictive contribution of all variables was statistically significant. With our final panel, we evaluated its performance using the MCCV process described above, and we also determined its in‐sample performance using a final prognostic model developed on all of the available data with LASSO with logistic regression. A cutoff was determined using the optimal Youdens index.

In all statistical analyses, a two‐tailed *P*‐value of <0.05 was considered statistically significant. All analyses were performed using the R statistical computing platform, version 3.4.4.

## 3 RESULTS

Forty‐three (4.8%) patients developed procedural AKI. Those who developed procedural AKI were older (70 vs 67 years of age, *P* = 0.04) and more likely to have prevalent diabetes mellitus (41.9% vs 23.5%, *P* = 0.01) or CKD (20.9% vs 10.4%, *P* = 0.04) (Table 1). Those who developed procedural AKI also had lower left ventricular ejection fraction at baseline (50.0% vs 56.6%, *P* = 0.04) and a higher percentage of them were prescribed an angiotensin‐converting enzyme inhibitor (ACEi)/angiotensin receptor blocker (ARB) compared to those who did not develop AKI (72.1% vs 53.6%, respectively, *P* = 0.02) (Table 1).

**Table 1:** Baseline characteristics of those who developed acute kidney injury compared to those who did not

Variable	With procedural AKI	Without procedural AKI	*P*
Age (years)	70 ± 11	67 ± 11	0.04
Male sex	31 (72.1%)	607 (71.7%)	1
Caucasian race	42 (97.7%)	785 (92.8%)	0.36
Body mass index (kg/m^2^)	28.7 ± 5.4	29.1 ± 5.6	0.67
Heart rate (beat/min)	70 ± 15	69 ± 14	0.67
Systolic blood pressure (mm Hg)	137 ± 30	136 ± 22	0.87
Diastolic blood pressure (mm Hg)	72 ± 11	72 ± 11	0.66
Smoker	4 (9.5%)	120 (14.3%)	0.50
Atrial fibrillation/flutter	8 (18.6%)	171 (20.2%)	1
Hypertension	37 (86.0%)	608 (71.9%)	0.05
Coronary artery disease	26 (60.5%)	431 (50.9%)	0.27
Prior myocardial infarction	13 (30.2%)	205 (24.2%)	0.37
Heart failure	12 (27.9%)	174 (20.6%)	0.25
Peripheral artery disease	13 (30.2%)	153 (18.1%)	0.07
Chronic obstructive pulmonary disease	11 (25.6%)	145 (17.2%)	0.15
Diabetes type I/type II	18 (41.9%)	199 (23.5%)	0.01
CVA/TIA	7 (16.3%)	85 (10.0%)	0.20
Chronic kidney disease	9 (20.9%)	88 (10.4%)	0.04
Prior angioplasty	6 (14.0%)	85 (10.0%)	0.43
Prior stent	17 (39.5%)	232 (27.4%)	0.12
Prior coronary artery bypass grafting	9 (20.9%)	163 (19.3%)	0.84
Prior percutaneous coronary intervention	16 (37.2%)	253 (29.9%)	0.31
ACEi/ARB	31 (72.1%)	451 (53.6%)	0.02
Beta blockers	27 (62.8%)	589 (69.8%)	0.40
Aldosterone antagonists	2 (4.7%)	30 (3.6%)	0.67
Loop diuretics	15 (34.9%)	180 (21.3%)	0.06
Nitrates	14 (32.6%)	166 (19.7%)	0.05
Calcium channel blockers	13 (30.2%)	193 (22.9%)	0.27
Statins	29 (67.4%)	612 (72.6%)	0.49
Aspirin	31 (72.1%)	643 (76.4%)	0.58
Warfarin	9 (20.9%)	127 (15.0%)	0.28
Clopidogrel	12 (27.9%)	188 (22.3%)	0.45
Left ventricular ejection fraction (%)	50 ± 18	57 ± 15	0.04
Sodium (mEq/L)	138.7 ± 3.4	139.3 ± 3.2	0.27
Blood urea nitrogen (mg/dL)	21 (16.5, 30)	18 (14, 23)	0.006
Blood urea nitrogen/creatinine	20.1 ± 6.9	17.8 ± 5.2	p = 0.04
Creatinine (mg/dL)	1.2 (0.9, 1.5)	1.1 (0.9, 1.3)	0.29
eGFR (CKD‐EPI) (mL/min/1.73 m^2^)	77.7 (63.8, 95.0)	99.2 (75.6, 110.7)	<0.001
Hemoglobin A1c	6.4 (6.2, 7.4)	6.1 (5.6, 6.9)	0.27
Hemoglobin (g/dL)	12.3 (1.5)	13.3 (1.7)	<0.001
C‐reactive protein (mg/L)	8.8 (3.8, 22.5)	3.5 (1.5, 9.1)	<0.001
CD5 antigen‐like (ng/mL)	3600 (2695, 5370)	3755 (2860, 5097.5)	0.77
Factor VII (ng/mL)	350 (290.5, 523)	468 (360, 588.75)	0.005
Osteopontin (ng/mL)	43 (31.5, 66)	27 (20, 41)	<0.001

AKI, acute kidney injury; ACEi/ARB, angiotensin converting enzyme inhibitor/angiotensin receptor blocker; CKD‐EPI, chronic kidney disease‐epidemiology; CVA/TIA, cerebrovascular accident/transient ischemic attack, eGFR, estimated glomerular filtration rate.

As expected, those who developed procedural AKI had higher blood urea nitrogen (BUN) (21 vs 18 mg/dL, *P* = 0.006) and BUN/creatinine ratio (20.1 vs 17.8, *P* = 0.04) and lower eGFR (77.7 vs 99.2 mL/min/1.73 m^2^, *P* < 0.001) and hemoglobin (12.3 vs 13.3 g/dL, *P* < 0.001) at baseline compared to those who did not develop procedural AKI. They also had higher baseline concentrations of C‐reactive protein (CRP) (8.8 vs 3.5 mg/L) and osteopontin (43 vs 27 ng/mL) and lower concentrations of Factor VII (350 vs 468 ng/mL) compared to those who did not develop procedural AKI (all significant *P*‐values). Those who developed procedural AKI had numerically lower concentrations of CD5 antigen‐like (3600 vs 3755 pg/mL) compared to those with did not develop procedural AKI (Table 1).

Following our machine learning‐driven approach to panel development, six predictors were present in the final model: four (history of diabetes, BUN/creatinine ratio, CRP, and osteopontin) had a positive association with AKI risk; while two (CD5 antigen‐like and Factor VII) had a negative association with AKI risk. Using the model‐building procedure described above for subsets of variables, the addition of each biomarker provided a statistically significant improvement in the AUC and the likelihood ratio, while decreasing the AIC and the BIC (Table 2).

**Table 2:** Procedural acute kidney injury risk score model calibration and goodness of fit

Panel	AIC	BIC	H‐L *P*
Diabetes	340.6	350.2	1
Diabetes + BUN/Cr	338.0	352.4	0.30
Diabetes + BUN/Cr + osteopontin	319.4	338.6	0.77
Diabetes + BUN/Cr + osteopontin + CRP	313.5	337.4	0.71
Diabetes + BUN/Cr + osteopontin + CRP + factor VII	309.1	337.8	0.77
Diabetes + BUN/Cr + osteopontin + CRP + factor VII + CD5 antigen‐like	305.0	338.5	0.96

AIC, akaike information criterion; BIC, Bayesian information criterion; BUN/Cr, blood urea nitrogen to creatinine ratio; CRP = C‐reactive protein; H‐L, Hosmer‐Lemeshow.

**Figure 1:**
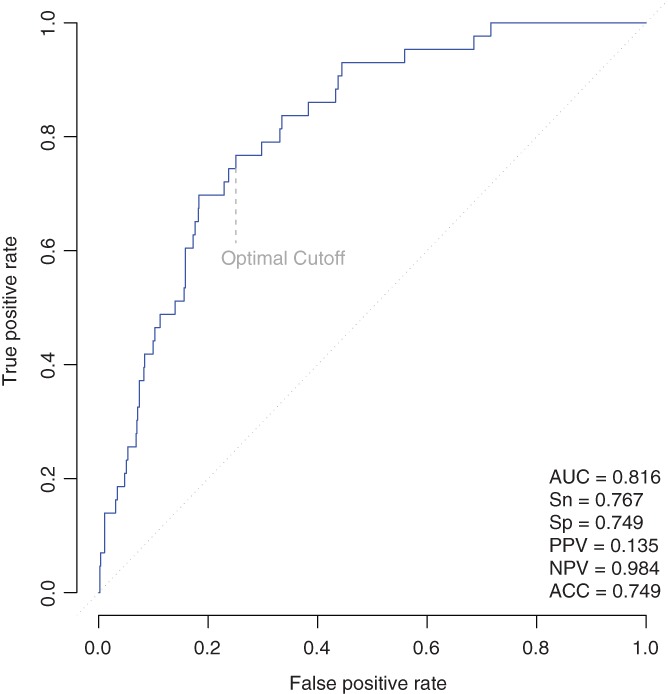
Procedural acute kidney injury risk prediction model receiver operating characteristic curve. ACC, accuracy; AUC, area under the receiver operating characteristic curve; NPV, negative predictive value; PPV, positive predictive value; Sn sensitivity, Sp, specificity,

The final model had a cross‐validated area under the receiver operating characteristic curve (AUC) of 0.79 for predicting procedural AKI, and an in‐sample AUC of 0.82 (*P* < 0.001). The optimal score cutoff had 77% sensitivity, 75% specificity, and a negative predictive value of 98% for procedural AKI (Figure 1). An elevated score was predictive of procedural AKI in all subjects (odds ratio = 9.87; *P* < 0.001). In addition, we tested our model in several subgroups and found that in women (n = 358) the AUC = 0.76; in those whose age ≥ 75 years (n = 285) the AUC = 0.81; in those with eGFR <60 (n = 181) the AUC = 0.87; in those with diabetes (n = 285) the AUC = 0.75; in those with HF (n = 205) the AUC = 0.86; and in those with PAD (n = 273) the AUC = 0.89.

## 4 DISCUSSION

Among a typical population of 889 patients undergoing coronary angiography with or without interventions for various acute and non‐acute indications, 4.8% of patients developed procedural AKI. We created a model that included six predictors of AKI: four (history of diabetes, BUN to creatinine ratio, CRP, and osteopontin) had a positive association with AKI risk; while two (CD5 antigen‐like and Factor VII) had a negative association with AKI risk. The final model had a high accuracy for predicting procedural AKI in patients undergoing coronary angiography.

The rationale for our study is based on the fact that AKI following coronary angiographic procedures is associated with significant morbidity and mortality that has potential to alter patient management if predicted early.^13^, ^14^ Ability to predict onset of AKI earlier might alter management in efforts toward its prevention, such as alteration of angiography plans (ie, minimizing dye exposure and employing bi‐plane angiography, for example), avoidance of nephrotoxins, or pre‐procedure hydration. In those at risk for CKD progression because the presence of comorbidities, such as diabetes and HF, interventions might be considered to reduce its incidence including lifestyle changes, better control of such comorbidities, avoidance of nephrotoxins, and consideration of delaying elective angiography plans until such comorbidities are better managed.

Prior work has examined this question, mostly based on clinical variables. Among patients in the Minnesota Registry of Interventional Cardiac Procedures, diabetes, increased age, higher dose and route of contrast administration, HF, hypertension, peri‐procedural shock, baseline anemia, post‐procedural drop in hematocrit, use of nephrotoxins, volume depletion, increased creatinine kinase‐muscle/brain enzyme, and need for cardiac surgery after contrast exposure were associated with increased risk of procedural AKI.^15^ Mehran et al developed a simple risk score that included pre‐ and peri‐procedural risk factors including hypotension, intra‐aortic balloon pump, HF, CKD, diabetes, age > 75 years, anemia, and volume of contrast with good discriminative power (c‐statistic 0.67).^4^ In another AKI risk prediction model developed by Brown et al, pre‐procedural serum creatinine, HF, and diabetes accounted for >75% of the predictive model.^16^, ^17^

While BUN and serum creatinine are most often used to predict procedural AKI, they are not very sensitive or specific for the diagnosis of AKI because they are affected by many renal and non‐renal factors that are independent of kidney injury or kidney function.^18^ As such, several biomarkers and biomarker panels with and without clinical risk factors have been examined to more accurately predict AKI. Our risk prediction model included the BUN/creatinine ratio in addition to clinical and biomarker risk factors to better predict procedural AKI. Given the proximity of collection of pre‐ and post‐procedure samples and the slower rise in creatinine, than BUN, it is understandable why the BUN and ratio of BUN/creatinine was predictive of renal dysfunction than creatinine alone.

Inflammation may play an important role in presence and severity of AKI. CRP is an acute‐phase protein of hepatic origin that is a marker of inflammation synthesized in response to factors released by macrophages and adipocytes.^19^ CRP has been associated with cardiovascular risk^20^ and has also been associated with renal dysfunction.^21^ Tang et al demonstrated that elevated serum CRP concentrations were associated with increased serum creatinine and urea concentrations (*P* < 0.01) in patients with AKI; CRP concentrations subsequently fell after recovery from AKI.^22^ In older patients with AKI, CRP was an independent risk factor for mortality.^23^ CRP has also been studied for its ability to predict risk for AKI. In a study of 1656 patients undergoing coronary artery bypass grafting, pre‐operative CRP concentrations predicted post‐operative AKI and mortality; the addition of CRP to an existing risk model improved net reclassification and discrimination.^24^ That we found concentrations of CRP as a predictor of procedural AKI is consistent with this body of evidence.

Osteopontin is an extracellular matrix protein and proinflammatory cytokine thought to facilitate the recruitment of monocytes/macrophages and to mediate cytokine secretion in leukocytes. It plays a role in many physiological and pathological processes, including biomineralization, tissue remodeling, and inflammation.^25^ It is found mainly in the loop of Henle and distal nephrons in normal kidneys and can be upregulated in all tubular and glomerular segments following kidney damage, and may also have a role in renal repair.^26^ In the last several years, the role of osteopontin in the pathogenesis of diabetic nephropathy has been explored.^25^ Osteopontin has been reported to be highly expressed in the tubular epithelium of the renal cortex and in glomeruli in rat and mouse models of diabetic nephropathy^27^ and in humans, plasma osteopontin concentrations are independently associated with the presence and severity of diabetic nephropathy.^28^ In a study of critically ill patients with AKI requiring renal replacement therapy, concentrations of osteopontin were significantly higher than in critically ill patients without AKI. In addition, osteopontin concentrations were found to be a strong predictor of mortality with an AUC of 0.82 (95% confidence interval [CI]: 0.74‐0.89; *P* < 0.0001), sensitivity of 100%,and specificity of 61% for a cutoff value of 577 ng/mL.^29^

CD5 antigen‐like is a secreted protein encoded by the *CD5L* gene that acts as a key regulator of lipid synthesis. It is mainly expressed by macrophages in lymphoid and inflamed tissues and regulates mechanisms in inflammatory responses, such as infection or atherosclerosis.^30^ Recently, in patients with diabetes, CD5 antigen‐like has been identified as a biomarker that may be able to improve rapid decline in kidney function independently of recognized clinical risk factors (odds ratio 0.52, 95% CI 0.29‐0.93) and improved model performance in predicting other indices of rapid eGFR decline.^31^

Data regarding Factor VII and its ability to predict kidney dysfunction are scarce; however, it is well established as a marker of hypercoagulability and persistence of inflammatory response.^32^ In a community‐based cohort of 588 elderly individuals, Fried et al found that elevations in CRP (*P* < 0.001), white blood count (*P* < 0.001), fibrinogen (*P* < 0.001), and Factor VII (*P* < 0.001) were associated with a subsequent rise in serum creatinine. Furthermore, CRP, white blood count, and Factor VII all independently predicted an eGFR decline of >3 mL/min/year/1.73 m^2^.^33^ In end‐stage renal disease (ESRD) patients bleeding diatheses is thought to be related to platelet dysfunction, vessel wall damage, and deficiencies in clotting factors II, VII, IX, and X; while the hypercoagulable state in ESRD is thought to be related to changes in the coagulation cascade, with increased levels of clotting factors VIIa, among others.^34^

Our AKI risk prediction model incorporated clinical and biomarker predictors all known to affect renal function and was based on an unbiased, machine learning approach for selection of model variables. Major advantages of our cohort are its detailed characterization and our experience working within this database, although limitations to our study exist. The CASABLANCA cohort was predominantly male, Caucasian, and representative of patients in a tertiary care referral center. In addition, we did not include the volume of contrast dye used during the coronary angiographic procedures, which clearly affects risk for AKI development or whether patients had prophylactic treatment for AKI prevention. In contrast to measures of kidney function (such as creatinine or eGFR), a theoretical advantage of our risk prediction model is the potential detection of AKI prior to change in measures of kidney function and the inclusion of several predictors associated with AKI development. Earlier prediction of AKI can allow for adjustments in patient/care management that might help to mitigate risk for severe kidney dysfunction.^35^ Nonetheless, data remain inconclusive regarding the role of adjunctive biomarker testing to support clinical decision making; our results are therefore noteworthy.

## 5 CONCLUSIONS

In a typical at‐risk population undergoing coronary angiography for various acute and non‐acute indications, we describe a clinical and proteomics‐supported biomarker model with high accuracy for predicting procedural AKI in patients undergoing coronary angiography. The ability to predict AKI may allow for earlier interventions in at‐risk patients to reduce future AKI risk. We plan to test our risk prediction model in an external validation cohort in the future.

## CONFLICTS OF INTEREST

Dr. Nasrien E. Ibrahim has received presentation fees from Novartis. Dr. Hanna K. Gaggin has received grant support from Roche Diagnostics and Jana Care, consulting income from Roche Diagnostics, and participates in clinical endpoint committees/data safety monitoring boards for Radiometer. Mr. Craig A. Magaret and Ms. Rhonda F. Rhyne are employed by Prevencio, Inc. Dr. James L. Januzzi has received grant support from Roche Diagnostics, Abbott, Singulex and Prevencio, consulting income from Roche Diagnostics, Critical Diagnostics, Janssen and Novartis, and participates in clinical endpoint committees/data safety monitoring boards for Novartis, Amgen, Pfizer, Janssen, AbbVie, and Boehringer‐Ingelheim. The other authors have nothing to disclose.

## Supporting information


**Table S1.** List of 109 biomarkers tested
