# Supplementary material for: A clinical, proteomics, and artificial intelligence‐driven model to predict acute kidney injury in patients undergoing coronary angiography
Source: Clin Cardiol. 2019 Jan 8;42(2):292–8. doi: 10.1002/clc.23143 (PMC6712314; doi:10.1002/clc.23143)
Supplement: Supplementary file 1 — Table S1. List of 109 biomarkers tested [file CLC-42-292-s001.docx]

**Supplemental Tables**

**Supplemental Table 1:** List of 109 biomarkers tested.

| **Biomarker** | **With Procedural**  **AKI** | **Without Procedural AKI** | **p** |
| --- | --- | --- | --- |
| Adiponectin (ug/mL) | 4.5 (2.6, 6.9) | 3.7 (2.4, 5.6) | 0.16 |
| Alpha-1-Antitrypsin (AAT) (mg/mL) | 2.2 (1.8, 2.6) | 1.8 (1.5, 2.1) | <0.001 |
| Alpha-2-Macroglobulin (A2Macro) (mg/mL) | 2.2 (1.7, 2.7) | 1.9 (1.5, 2.3) | 0.05 |
| Angiopoietin-1 (ANG-1) (ng/mL) | 7.5 (5.4, 12) | 6.8 (4.9, 10) | 0.24 |
| Angiotensin-Converting Enzyme (ACE) (ng/mL) | 82 (58.5, 104.5) | 79 (61.3, 104.8) | 1.00 |
| Apolipoprotein(a) (Lp(a)) (ug/mL) | 197 (62, 443) | 202.5 (69.3, 493.8) | 0.64 |
| Apolipoprotein A-I (Apo A-I) (mg/mL) | 1.8 (1.5, 2.2) | 1.8 (1.5, 2.2) | 0.95 |
| Apolipoprotein A-II (Apo A-II) (ng/mL) | 297 (269, 354) | 313.5 (252, 385) | 0.35 |
| Apolipoprotein B (Apo B) (ug/mL) | 1190 (899, 1645) | 1410 (1090, 1860) | 0.005 |
| Apolipoprotein C-I (Apo C-I) (ng/mL) | 307 (274.5, 361) | 317.5 (260, 380) | 0.93 |
| Apolipoprotein C-III (Apo C-III) (ug/mL) | 211 (173.5, 255.5) | 215 (159, 268.8) | 0.87 |
| Apolipoprotein H (Apo H) (ug/mL) | 312 (266.5, 368) | 331 (271.3, 389.8) | 0.34 |
| Beta-2-Microglobulin (B2M) (ug/mL) | 2.1 (1.8, 3) | 1.7 (1.4, 2.3) | <0.001 |
| Brain-Derived Neurotrophic Factor (BDNF) (ng/mL) | 2.6 (1.3, 4.2) | 2.3 (1, 4.7) | 0.43 |
| C-Reactive Protein (CRP) (ug/mL) | 8.8 (3.8, 22.5) | 3.5 (1.5, 9.1) | <0.001 |
| Calbindin (ng/mL) | 8 (8, 20) | 8 (8, 8) | p = 0.01 |
| Carbonic anhydrase 9 (CA-9) (ng/mL) | 0.2 (0.1, 0.3) | 0.14 (0.1, 0.2) | 0.11 |
| Carcinoembryonic antigen-related cell adhesion molecule 1 (CEACAM1) (ng/mL) | 25 (22, 30.5) | 23 (20, 27) | 0.07 |
| CD5 Antigen-like (CD5L) (ng/mL) | 3600 (2695, 5370) | 3755 (2860, 5097.5) | 0.77 |
| Decorin (ng/mL) | 3.3 (2.2, 4.4) | 2.3 (1.9, 3.4) | 0.004 |
| E-Selectin (ng/mL) | 4.8 (3.3, 6.1) | 5.2 (3.7, 7) | 0.21 |
| EN-RAGE (ng/mL) | 39 (18, 57) | 27 (17, 48) | 0.09 |
| Eotaxin-1 (pg/mL) | 98 (65.3, 154.5) | 95.5 (42.5, 141) | 0.24 |
| Factor VII (ng/mL) | 350 (290.5, 523) | 468 (360, 588.8) | 0.005 |
| Fatty Acid-Binding Protein, heart (FABP, heart) (ng/mL) | 4.6 (4.6, 10.4) | 4.6 (4.6, 4.6) | <0.001 |
| Ferritin (FRTN) (ng/mL) | 139 (70.5, 204) | 134 (72.3, 232) | 0.96 |
| Fetuin-A (ug/mL) | 568 (483, 777.5) | 698 (588.3, 829) | 0.003 |
| Fibrinogen (mg/mL) | 5.2 (4.2, 6.1) | 4.4 (3.6, 5.4) | 0.002 |
| Follicle-Stimulating Hormone (FSH) (mIU/mL) | 7.2 (3.7, 38) | 6.8 (3.7, 28) | 0.72 |
| Glucagon-like Peptide 1, total (GLP-1 total) (pg/mL) | 3.5 (3.5, 3.5) | 3.5 (3.5, 3.5) | 0.44 |
| Granulocyte-Macrophage Colony-Stimulating Factor (GM-CSF) (pg/mL) | 10.5 (10.5, 10.5) | 10.5 (10.5, 10.5) | 0.75 |
| Growth Hormone (GH) (ng/mL) | 0.5 (0.2, 1.4) | 0.3 (0.1, 0.9) | 0.07 |
| Haptoglobin (mg/mL) | 1.5 (0.9, 2.3) | 1.2 (0.6, 1.9) | 0.05 |
| Immunoglobulin A (IgA) (mg/mL) | 2.6 (1.8, 3.7) | 2.4 (1.6, 3.4) | 0.27 |
| Immunoglobulin M (IgM) (mg/mL) | 1.2 (0.9, 1.6) | 1.4 (0.9, 2.1) | 0.13 |
| Insulin (uIU/mL) | 1.1 (0.3, 2.3) | 0.8 (0.1, 2.1) | 0.30 |
| Intercellular Adhesion Molecule 1 (ICAM-1) (ng/mL) | 112 (86.5, 132.5) | 104 (85, 130) | 0.45 |
| Interferon gamma (IFN-gamma) (pg/mL) | 1.3 (1.3, 1.3) | 1.3 (1.3, 1.3) | 0.003 |
| Interferon gamma Induced Protein 10 (IP-10) (pg/mL) | 311 (256, 466) | 304 (233, 402.8) | 0.20 |
| Interleukin-1 alpha (IL-1 alpha) (ng/mL) | 0.001 (0.001, 0.001) | 0.001 (0.001, 0.001) | 0.31 |
| Interleukin-1 beta (IL-1 beta) (pg/mL) | 3.3 (3.3, 8.5) | 6.6 (3.3, 8.4) | 0.84 |
| Interleukin-1 receptor antagonist (IL-1ra) (pg/mL) | 118 (68.5, 162) | 114 (87.3, 144) | 0.90 |
| Interleukin-2 (IL-2) (pg/mL) | 20.5 (20.5, 20.5) | 20.5 (20.5, 20.5) | 0.75 |
| Interleukin-3 (IL-3) (ng/mL) | 0.003 (0.003, 0.003) | 0.003 (0.003, 0.003) | 0.75 |
| Interleukin-4 (IL-4) (pg/mL) | 17.5 (17.5, 17.5) | 17.5 (17.5, 17.5) | 0.75 |
| Interleukin-5 (IL-5) (pg/mL) | 2.4 (2.4, 2.4) | 2.4 (2.4, 2.4) | 0.12 |
| Interleukin-6 (IL-6) (pg/mL) | 2.3 (2.3, 2.3) | 2.3 (2.3, 2.3) | 0.05 |
| Interleukin-6 receptor (IL-6r) (ng/mL) | 25 (18.5, 31) | 24 (19, 29) | 0.76 |
| Interleukin-7 (IL-7) (pg/mL) | 16 (16, 16) | 16 (16, 16) | 0.75 |
| Interleukin-8 (IL-8) (pg/mL) | 8.2 (6.15, 13.5) | 6.4 (4.4, 9.8) | 0.003 |
| Interleukin-10 (IL-10) (pg/mL) | 3.4 (3.4, 3.4) | 3.4 (3.4, 3.4) | 0.74 |
| Interleukin-12 Subunit p40 (IL-12p40) (ng/mL) | 0.6 (0.4, 0.7) | 0.6 (0.5, 0.7) | 0.45 |
| Interleukin-12 Subunit p70 (IL-12p70) (pg/mL) | 25 (25, 25) | 25 (25, 25) | 0.003 |
| Interleukin-15 (IL-15) (ng/mL) | 0.5 (0.2, 0.7) | 0.6 (0.5, 0.7) | 0.52 |
| Interleukin-17 (IL-17) (pg/mL) | 1.5 (1.5, 1.5) | 1.5 (1.5, 1.5) | 0.26 |
| Interleukin-18 (IL-18) (pg/mL) | 191 (140, 264) | 200 (149, 268) | 0.90 |
| Interleukin-18-binding protein (IL-18bp) (ng/mL) | 11 (8.8, 17) | 9.2 (7.1, 12) | 0.003 |
| Interleukin-23 (IL-23) (ng/mL) | 2.1 (1.7, 3.1) | 2.5 (2, 3.2) | 0.23 |
| Kidney Injury Molecule-1 (KIM-1) (ng/mL) | 0.05 (0.03, 0.1) | 0.04 (0.01, 0.06) | 0.004 |
| Lectin-Like Oxidized LDL Receptor 1 (LOX-1) (ng/mL) | 0.3 (0.3, 0.7) | 0.3 (0.3, 0.3) | 0.04 |
| Leptin (ng/mL) | 8.5 (5.3, 20.5) | 8.9 (4.5, 20) | 0.94 |
| Luteinizing Hormone (LH) (mIU/mL) | 5.7 (3.1, 11) | 4.8 (3.3, 9.8) | 0.52 |
| Macrophage Colony-Stimulating Factor 1 (M-CSF) (ng/mL) | 0.7 (0.4, 1.3) | 0.4 (0.2, 0.6) | <0.001 |
| Macrophage Inflammatory Protein-1 alpha (MIP-1 alpha) (pg/mL) | 14.5 (14.5, 37.5) | 14.5 (14.5, 35) | 0.89 |
| Macrophage Inflammatory Protein-1 beta (MIP-1 beta) (pg/mL) | 278 (225.5, 391) | 268 (195, 361) | 0.16 |
| Macrophage Inflammatory Protein-3 alpha (MIP-3 alpha) (pg/mL) | 10 (10, 29.5) | 10 (10, 26) | 0.41 |
| Matrix Metalloproteinase-1 (MMP-1) (ng/mL) | 0.3 (0.3, 0.3) | 0.3 (0.3, 0.3) | 0.78 |
| Matrix Metalloproteinase-2 (MMP-2) (ng/mL) | 1490 (1300, 1825) | 1310 (1110, 1610) | 0.002 |
| Matrix Metalloproteinase-3 (MMP-3) (ng/mL) | 7.5 (5.7, 14.5) | 6.6 (4.7, 9.8) | 0.06 |
| Matrix Metalloproteinase-7 (MMP-7) (ng/mL) | 0.4 (0.2, 0.6) | 0.4 (0.2, 0.5) | 0.83 |
| Matrix Metalloproteinase-9 (MMP-9) (ng/mL) | 126 (92.5, 181.5) | 120 (86, 170) | 0.46 |
| Matrix Metalloproteinase-9, total (MMP-9, total) (ng/mL) | 626 (430.5, 916) | 545 (399.3, 775) | 0.14 |
| Matrix Metalloproteinase-10 (MMP-10) (ng/mL) | 0.1 (0.1, 0.1) | 0.1 (0.1, 0.1) | 0.58 |
| Midkine (ng/mL) | 18 (11, 27) | 14 (9.9, 19) | 0.01 |
| Monocyte Chemotactic Protein 1 (MCP-1) (pg/mL) | 127 (88, 161) | 110 (76, 161) | 0.23 |
| Monocyte Chemotactic Protein 2 (MCP-2) (pg/mL) | 26 (17, 34) | 23 (18, 29) | 0.26 |
| Monocyte Chemotactic Protein 4 (MCP-4) (pg/mL) | 2200 (1650, 3260) | 2280 (1620, 3397.5) | 0.93 |
| Monokine Induced by Gamma Interferon (MIG) (pg/mL) | 1230 (829, 1830) | 915.5 (578, 1607.5) | 0.04 |
| Myeloid Progenitor Inhibitory Factor 1 (MPIF-1) (ng/mL) | 1.4 (1.2, 2) | 1.2 (0.95, 1.5) | 0.008 |
| Myoglobin (ng/mL) | 35 (24, 56) | 32 (22, 46) | 0.11 |
| N-terminal pro B-type natriuretic peptide (NT proBNP) (pg/mL) | 4490 (1780, 15975) | 1445 (516.8, 3762.5) | <0.001 |
| Osteopontin (ng/mL) | 43 (31.5, 66) | 27 (20, 41) | <0.001 |
| Pancreatic Polypeptide (PPP) (pg/mL) | 115 (63, 201) | 84 (48, 158) | 0.05 |
| Plasminogen Activator Inhibitor 1 (PAI-1) (ng/mL) | 43 (27.5, 76.5) | 46 (27, 72.8) | 0.87 |
| Platelet endothelial cell adhesion molecule (PECAM-1) (ng/mL) | 55 (46, 68.5) | 54 (45, 63) | p = 0.44 |
| Prolactin (PRL) (ng/mL) | 8.6 (5.9, 13) | 8.1 (5.5, 12.8) | 0.33 |
| Pulmonary and Activation-Regulated Chemokine (PARC) (ng/mL) | 114 (79, 173) | 100 (74.3, 137) | 0.16 |
| Pulmonary surfactant-associated protein D (SP-D) (ng/mL) | 6.8 (4.6, 9.9) | 5.1 (3.3, 8.3) | 0.02 |
| Resistin (ng/mL) | 2.8 (2.0, 3.8) | 2.4 (1.8, 3.4) | 0.13 |
| Serotransferrin (Transferrin) (mg/dl) | 276 (232.5, 308.5) | 272 (234, 315) | 0.98 |
| Serum Amyloid P-Component (SAP) (ug/mL) | 12 (9, 15.5) | 13 (10, 16) | 0.21 |
| Stem Cell Factor (SCF) (pg/mL) | 400 (309, 502) | 362 (279, 449.8) | 0.09 |
| T-Cell-Specific Protein RANTES (RANTES) (ng/mL) | 11 (6.2, 18.5) | 8.5 (3.9, 18) | 0.17 |
| Tamm-Horsfall Urinary Glycoprotein (THP) (ug/mL) | 0.03 (0.02, 0.04) | 0.03 (0.02, 0.04) | 0.02 |
| Thrombomodulin (TM) (ng/mL) | 3.8 (3.2, 5.05) | 3.8 (3.1, 4.6) | 0.35 |
| Thrombospondin-1 (ng/mL) | 4450 (3050, 7595) | 4625 (2160, 7622.5) | 0.52 |
| Thyroid-Stimulating Hormone (TSH) (uIU/mL) | 1.3 (0.7, 2.2) | 1.2 (0.8, 1.9) | 0.79 |
| Thyroxine-Binding Globulin (TBG) (ug/mL) | 35 (31, 42.5) | 38 (31, 45) | 0.12 |
| Tissue Inhibitor of Metalloproteinases 1 (TIMP-1) (ng/mL) | 92 (72, 117.5) | 72 (59, 90) | <0.001 |
| Transthyretin (TTR) (mg/dl) | 21 (18, 25.5) | 26 (21, 30) | 0.002 |
| Tumor Necrosis Factor alpha (TNF-alpha) (pg/mL) | 6.5 (6.5, 6.5) | 6.5 (6.5, 6.5) | <0.001 |
| Tumor Necrosis Factor beta (TNF-beta) (pg/mL) | 20 (20, 20) | 20 (20, 20) | 1.00 |
| Tumor necrosis factor receptor 2 (TNFR2) (ng/mL) | 8.1 (5.75, 11) | 6.3 (4.8, 8.7) | 0.003 |
| Vascular Cell Adhesion Molecule-1 (VCAM-1) (ng/mL) | 628 (488.5, 843) | 563.5 (456, 706) | 0.03 |
| Vascular Endothelial Growth Factor (VEGF) (pg/mL) | 86 (70.5, 145) | 98 (68, 135) | 0.97 |
| Vitamin D-Binding Protein (VDBP) (ug/mL) | 243 (193, 288.5) | 249 (184, 313) | 0.50 |
| Vitamin K-Dependent Protein S (VKDPS) (ug/mL) | 13 (9.8, 17.5) | 14 (11, 16.8) | 0.28 |
| Vitronectin (ug/mL) | 407 (341.5, 506.5) | 462 (351, 572.8) | 0.06 |
| von Willebrand Factor (vWF) (ug/mL) | 164 (132, 202.5) | 131 (95.3, 182) | 0.002 |

AKI = acute kidney injury.
